# Bone marrow micro-environment is a crucial player for myelomagenesis and disease progression

**DOI:** 10.18632/oncotarget.14610

**Published:** 2017-01-12

**Authors:** Patrizia Mondello, Salvatore Cuzzocrea, Michele Navarra, Michael Mian

**Affiliations:** ^1^ Department of Human Pathology, University of Messina, Messina, Italy; ^2^ Department of Chemical, Biological, Pharmaceutical and Environmental Sciences, University of Messina, Messina, Italy; ^3^ Lymphoma Service, Memorial Sloan Kettering Cancer Center, New York, NY, USA; ^4^ Department of Hematology and Center of Bone Marrow Transplantation, Hospital of Bolzano, Bolzano/Bozen, Italy; ^5^ Department of Internal Medicine V, Hematology & Oncology, Medical University Innsbruck, Innsbruck, Austria

**Keywords:** multiple myeloma, micro-environment, therapeutic opportunities, osteoclast activation, angiogenesis

## Abstract

Despite the advent of many therapeutic agents, such as bortezomib and lenalidomide that have significantly improved the overall survival, multiple myeloma remains an incurable disease. Failure to cure is multifactorial and can be attributed to the underlying genetic heterogeneity of the cancer and to the surrounding micro-environment. Understanding the mutual interaction between myeloma cells and micro-environment may lead to the development of novel treatment strategies able to eradicate this disease. In this review we discuss the principal molecules involved in the micro-environment network in multiple myeloma and the currently available therapies targeting them.

## INTRODUCTION

Multiple myeloma (MM) is a malignant lymphoproliferative disorder deriving from a clonal plasma cell dyscrasia with the ability to produce monoclonal immunoglobulins in most cases. The incidence of the disease has been estimated to be 26,850 new cases in 2015 in the United States. Disease occurrence increases with age, affecting mostly people in the sixth and seventh decade of life with a predominance in males and Afro-Americans [1]. Clinical presentation at onset is highly variable ranging from a completely asymptomatic disease without evidence of end-organ damage to an acute life threatening condition.

**Figure 1:**
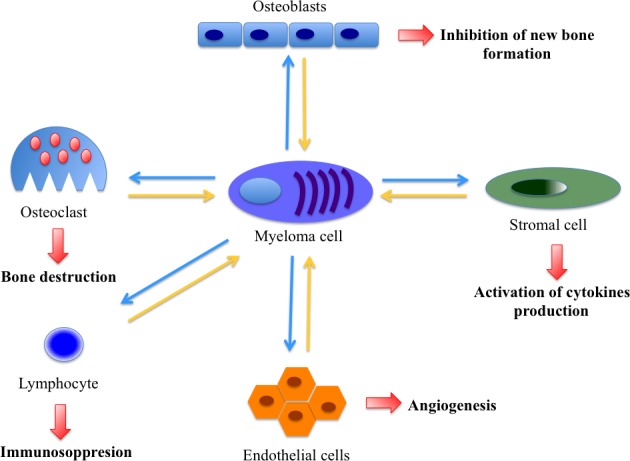
Interplay between various micro-environmental cells promoting angiogenesis and proliferation in multiple myeloma

Despite intensive research, the etiology of MM is largely unknown. Possible risk factors include exposure to ionizing radiation, chemicals such as benzene, asbestos, lucite and antigens. Also genetic factors play a major role [2]. Transformation of a normal plasma cell into a neoplastic cell is a multistep process [3] due to genetic and molecular events [4] as well as to important and irreversible alterations in the bone marrow micro-environment [5, 6]. The myeloma cell is immersed in the bone marrow micro-environment where it mutually interacts with stromal cells (BMSC), osteoblasts, osteoclasts, lymphocytes and endothelial cells [6] (Figure 1). Indeed, culture of plasma cells is only possible in the presence of BMSC [6].

A process of major importance is the interaction between myeloma cells and BMSC through adhesion molecules, such as VLA-4 and LFA-1. These interactions trigger a complex cytokine network between cancer cells and micro-environment, ultimately impacting further development and prognosis of the disease itself. Another system interaction involves *C-X-C chemokine ligand* (CXCL)-12, expressed by BMSC, and its receptor *C-X-C chemokine receptor* (CXCR)-4, located on MM cells, which is involved in motility and cytoskeletal rearrangements [6]. Moreover, CXCL-12 up-regulates transiently VLA-4, further influencing cellular adhesion of myeloma cells to the BMSC and cytokines secretion [7]. However, adhesion molecules are not the only players in this complex game: pathogenesis of MM also depends on the presence of growth factors [8, 9] that are usually produced by BMSC to regulate activity of lympho-hemopoietic cells [10, 11].

In the present review, we focus on the pathogenetic mechanisms involving the bone marrow micro-environment and promoting myeloma.

## INTERLEUKINE 6 (IL-6)

IL-6 is produced by mononuclear phagocytes, endothelial cells, fibroblasts and many other cell types as a response to IL-1 and tumor necrosis factor (TNF). This molecule also stimulates the secretion of the proteins of the acute phase immune response, such as the protein or mannose-binding fibrinogen by the liver cells. IL-6 acts as a growth factor for activated B-cells and differentiation towards the plasma cell line and has multiple effects on hematopoietic and other cells [12]. It is closely involved in the pathogenesis of MM: 1) IL-6 induces *in vitro* growth of fresh cells isolated from myeloma patients; 2) The myeloma cells spontaneously produce IL-6 and express the corresponding receptor; 3) antibodies against IL-6 inhibit the growth of myeloma cells; 4) treatment of myeloma patients with antibodies against IL-6 has shown anti-tumor effect [9, 13, 14]; 5) retinoic acid induces apoptosis in myeloma cells by down-regulation expression of the IL-6 receptor [15]. Preliminary data suggests that the secretion of IL-6 is regulated by plasmoblast cytokines, such as TNF-alpha and transforming growth factor (TGF)-beta [9].

Enhancing sensitivity of the myeloma cell to IL-6 contributes to the growth and expansion of the neoplastic cells, as is the case with the soluble receptors for IL-6 (sIL-6R) [16]. These receptors derive from cleavage of the receptor itself or from alternative splicing mechanisms of the respective RNA [17, 18]. sIL-6R is present in the serum and urine of healthy individuals [9], but it is significantly elevated in MM patients [16, 19–22]. Therefore, the significance of sIL -6R is controversial. Unlike other Authors [16, 20, 22], Ohtani et al. [19] observed a good correlation between sIL-6R levels and tumor burden. This is in line with the observation that elevated serum levels of IL-6 as well as its soluble receptor are able to predict a poor prognosis and to reflect the level of disease activity [16, 23, 24], whereas the decrease of these parameters is associated with a good response to treatment [20].

Interaction between MM cell and BMSCs stimulates IL-6 secretion [25]. Originally identified as a regulator of normal B-cell differentiation, IL-6 has shown to promote myeloma cell proliferation and protect cells from apoptosis [26]. After co-culture with BMSCs, MM cells increased levels of phosphorylated AKT and ERK [27, 28], cyclin D2, CDK4, and Bcl-XL, and decreased cleaved Caspase- 3 and PARP [29], which are important signaling pathways involved in proliferation and apoptosis of MM cells.

Furthermore, IL-6 contributes to the dysfunction of immunosystem. In MM patients dendritic cells (DCs) presented a lower expression of HLA-DR, CD40 and CD80 antigens, and impaired activation of T-cell proliferation compared with controls. These DCs were unable to present the specific tumor antigen to autologous T cells [30]. Hwang et al demonstrated that the IL-6Rα knockdown-DC vaccine significantly enhances the frequency of tumor-specific CD8+ producing effector molecules such as IFN-γ, TNF-α, FasL, perforin, and granzyme B, and generates more memory T cells, resulting in prolonged survival [31].

Finally, IL-6 is also produced by osteoclasts. These cells produce high levels of IL-6 when grown in co-culture with MM cells, resulting in further increase of cell proliferation and inhibition of apoptosis [32, 33]. Although its precise role is still under debate, IL-6 released by osteoclasts seems to increase MM tumor burden, and enhance bone destruction since it increases production of IL-17 by T-cells. Increased IL-17 secretion by these bone marrow T cells results in up-regulation of RANKL and increases osteoclast formation [34]. Expression of the IL-17 receptor on MM plasma cells leads to IL-17 mediated growth of plasma cells [35].

Overall, therapies against IL-6 would be a rational target. Siltuximab is a monoclonal antibody (Ab) against IL-6, which has demonstrated to have a good safety profile (NCT01484275) [36]. Therefore, it was evaluated in transplant-ineligible MM patients in combination with bortezomib, melphalan, and prednisone (VMP). However, Siltuximab did not demonstrate an improvement in terms of complete response rate when compared to the VMP alone [37]. Since IL-6 is involved in early myeloma-stroma interaction and survival of neoplastic cells, IL-6 therapy at earlier stages of the disease might be more beneficial. Therefore, Siltuximab is currently under investigation in patients with asymptomatic disease (NCT01484275).

## OSTEOCLAST ACTIVATION

Osteoclast activation contributes to the decrease of bone matrix and ultimately to osteolysis. In normal conditions, osteoclast differentiation and activation are controlled by stromal cell/osteoblast through the Receptor Activator of NF-κB Ligand (RANK-L)/RANK/osteoprotegerin (OPG) system [38–41]. Osteocytes are the major source of the osteoclastogenic cytokine RANK-L [42, 43]. The specific receptor of this protein, RANK, is located on osteoclast progenitors and its stimulation leads to differentiation into mature and active osteoclasts [40, 41, 44]. RANK-L activity is physiologically countered by interferon-gamma (INFγ), which regulates osteoclast formation by preventing excessive bone resorption [39–41].

**Figure 2:**
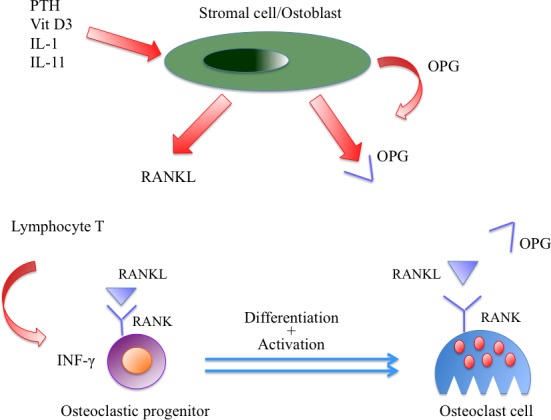
Bone remodelling in multiple myeloma Myeloma cells also directly promote osteoclast formation *via* the endogenous expression of RANKL, and downregulation of the RANKL decoy receptor (OPG).

In MM, RANK expression is dysregulated in osteoclast precursor cells [45]. Myeloma cells also directly promote osteoclast formation *via* the endogenous expression of RANK-L [45], TNF-α, and macrophage inflammatory protein 1 alpha (MIP-1α) [46–50]. Myeloma cells further contribute to osteoclastogenesis by downregulating expression of the RANK-L decoy receptor (OPG) [51, 52]. (Figure 2) This inhibitory effect is secondary to the interaction of myeloma cells and BMSC through VLA4 and VCAM1 [47, 53]. Preclinical studies have supported the importance of the RANK-L/OPG in the pathogenesis of bone lesions in MM. In xenograft model, treatment with either OPG or inhibitor of the receptor RANK inhibited bone lysis and reduced the osteoclasts number [54]. Furthermore, inhibition of this receptor resulted in decreasing of tumor burden and paraprotein levels in MM. Elevated levels of IL-6 induce RANK-L expression also in lymphocytes and decrease INFγ production [55], leading to bone resorption. Ultimately, there is an imbalance of osteoclast homeostasis in favor of RANK-L and to the detriment of OPG and INFγ, resulting in osteoclast activation, bone resorption and myeloma cell survival [56].

Therefore, bisphosphonates and monoclonal antibodies binding RANK-L are commonly used to limit osteoclast activity [57, 58]. These therapies significantly delay skeletal-related events such as pathological fracture and therefore reduce disease-associated morbidity [59, 60], and prolong survival [61–63].

Osteoblast-mediated bone formation/mineralization is significantly reduced in MM patients, contributing to bone destruction. The factors involved in osteoblast suppression have been widely described within the Wnt pathway and myeloma-derived factors [64]. Many therapeutic agents that increase bone formation or block agonists in myeloma patients are currently under investigation. These include potent osteogenic factors such as Wnt, Dickkopf Wnt signaling pathway 1 (DKK-1), fibroblast growth factor (FGF) 23, and heparanase [65–67]. In xenograft model, the DKK-1 inhibitor BHQ880 increased osteoblast number and trabecular bone volume [68]. Similarly, using this drug combined with bisphosphonate resulted in a trend towards increased bone mineral density in relapsed MM [69]. Other phase I/II clinical trials examining its efficacy are completed with pending reports (NCT01302886, NCT00741377, and NCT01337752).

## CD28

CD8+ T cells recognize and eliminate cells infected by intracellular pathogens [70] as well as neoplastic cells [71]. The T-cell receptor (TCR) identifies such cells by interaction with MHC-I-receptor and the bound peptide antigen presented on the surface of antigen-presenting cells (APCs), namely DCs and macrophages/monocytes [72]. However, the stimulation *via* TCR alone is not sufficient to activate the CD8+ T cells [73–75] since a co-stimulatory signal is necessary [75]. One of the best characterized co-stimulus is the interaction of CD28 of the T cell and CD86 or CD80 of the APC [72, 73]. Persistent antigenic stimulation or repetitive stimulation/proliferation rounds lead to progressively down-regulation of CD28 expression and accumulation of highly antigen experienced CD8+ CD28 T cells [72, 74]. The decrease of CD28 is associated with the increased expression of CD57 (HNK, Leu-7, L-2) [76, 77], a terminally sulphated carbohydrate-determinant found on various surface glycoproteins, proteoglycans and glycolipids on subsets of natural killer (NK) cells, T cells, and others [72, 78, 79].

High levels of CD8+ CD28- (CD8+ CD57+) T-cell population are associated with malignancy. Increased number of these T cells was found both in the micro-enviroment and peripheral blood of patients with solid tumors [71, 80–83] and hematologic malignancies [84–88]. Patients affected by melanoma showed the expanded CD8+ CD28- T cells with high perforin expression, suggesting an active immune response against the tumor [81]. Conversely, lung cancer patients presented expanded CD8+ CD28- T cells with significant levels of FOXP3+, which were attributed to the immunosuppressive component of the antitumour immune response [82]. Probably the predominance of cytotoxic *versus* immunosuppressive CD8+ CD28- T cell population differs in various types of cancer or even between individual patients with the same oncological disease.

## CD38

CD38 is a multifunctional cell surface protein that has receptor [89] as well as enzyme functions [90, 91] with an important role in cell signaling. This protein is found at high levels on normal plasma cells as well as in hematological malignancies [92–98]. CD38 levels are even higher in malignant plasma cells [99], which is why it is an attractive therapeutic target for MM. Various anti-CD38 antibodies have been developed and up to now available preclinical and clinical data are very promising. The so far best investigated antibody is Daratumumab. This IgG1 antibody binds a unique epitope present on the CD38 molecule leading to complement-dependent cytotoxicity [100]. Furthermore, Daratumumab decreases the CD38+ immunosopressive cellular population, increases T-helper and cytotoxic T-cells population, and enhances TCR clonality [101]. *In vivo* daratumumab alone showed substantial tumor cell lysis. This effect markedly increased, when the antibody was combined with anti-myeloma drugs, such as lenalidomide and bortezomib [102, 103]. Its clinical efficacy was evaluated in the CASTOR and Pollux trial [104, 105]. The former included nearly 500 relapsed or refractory multiple myeloma patients, each of whom underwent eight cycles of a standard two-drugs induction regimen (bortezomib and dexamethasone). Afterwards they were randomized to receive daratumumab maintenance therapy or not. In interim analysis, daratumumab combination reduced the risk of cancer progression by 70%, and doubled both rates of very good partial response from 29% to 59% and complete response from 9% to 19% with a manageable toxicity profile [104]. Also in the POLLUX trial, in which 569 relapsed or refractory patients were treated with daratumumab in combination with lenalidomide and dexamethasone, the addition of the antibody significantly prolonged progression-free survival (PFS) (*p* < 0.0001) and reduced the risk of disease progression of 63% [105]. Based on these promising results, Daratumumab was approved by the U.S. Food and Drug Administration as the first monoclonal antibody for the treatment of multiple myeloma.

Currently, there are ongoing phase 3 studies, which include daratumumab in combination with either lenalidomide or bortezomib-based regimens in newly diagnosed MM (NCT02252172, NCT02195479, and NCT02541383). An additional phase 1 study is also investigating the subcutaneous delivery of daratumumab in association with recombinant human hyaluronidase in patients with relapsed/refractory MM (NCT02519452).

## PD-1 AND PD-L1

**Figure 3:**
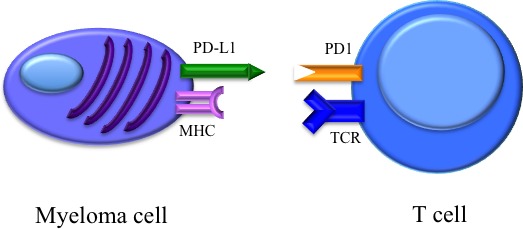
Binding of PD-1 on the T cell with tumor-associated PD-L1 results in downregulation of T-cell effector functions

Tumors can escape immunosurveillance by expression of molecules that inhibit antitumor immunoresponse, such as programmed cell death ligand 1 (PD-L1) [106]. Although PD-L1 expression has not been observed in normal epithelial cells, it is highly expressed on many solid tumors [107]. PD-1 is a cell surface receptor of the immunoglobulin superfamily and is expressed on T cells, B cells, and NK cells [108]. Within the tumor, cells of the micro-environment express PD-L1, leading to T cell anergy upon cellular contact. (Figure 3) Furthermore, T cells produce INFγ, which upregulates PD-L1 expression on tumor and infiltrating immune cells, forming a feedback loop that generates a PD-1 signal maintaining immunosuppression [109, 110]. Although not detected on normal plasma cells, myeloma cell lines and primary myeloma cells up-regulate PD-L1, while its ligand PD-1 is found on a proportion of T-cells in myeloma patients [111–115]. The highest PD-L1 expression levels were detected in relapsed/refractory MM and correlated with tumor burden and poor treatment response [107]. Anti-PD-L1 antibodies inhibit dendritic cells and myeloid-derived suppressor cells enhancing the cytolytic activity of NK cells against MM cell [114]. Phase I studies using checkpoint inhibitors reported disappointing results, achieving stable disease as best response [116, 117]. Nevertheless, combination treatments including these checkpoint inhibitors could lead to an increased anti-MM host immunity and therefore better clinical responses. Clinical trials investigating the combination of anti-PD-L1 treatment with either immunomodulatory agents, such as lenalidomide (NCT02077959), myeloma vaccines (e.g., NCT01067287), or other T-cell co-inhibitor molecules such as cytotoxic T-lymphocyte-associate protein 4 (NCT01592370), which modulate MM-host immune responses, are ongoing.

## ANGIOGENESIS AND ANGIOGENIC FACTORS

**Figure 4:**
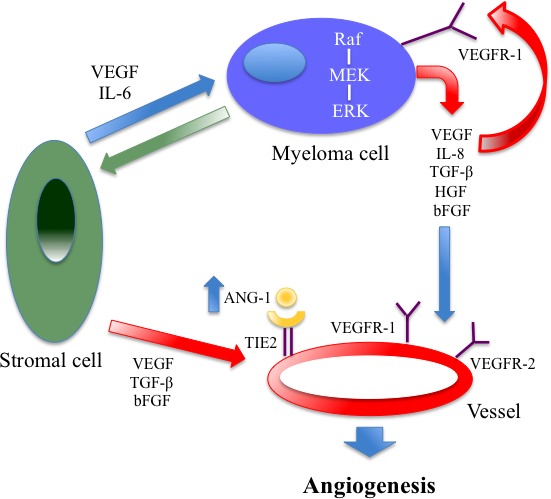
Autocrine and paracrine VEGF-mediated pathways in multiple myeloma: both are important for tumor angiogenesis and growth A close relationship between VEGF and IL-6 has been found in the paracrine pathways.

In 1999 Vacca et al described an increased neovascularization of the bone marrow stroma in patients affected by MM [118]. The grade of this neovascularization seems to increase during the evolution from monoclonal gammopathy of undetermined significance (MGUS) to MM [119]. This process is triggered by neoangiogenetic factors, which are produced from the BMSC, [such as vascular endothelial growth factor (VEGF), basic fibroblasr growth factor (b-FGF) and TGF-β, and from the myeloma cells themselves (such as VEGF, IL-8 and TGF-β). (Figure 4)

### Vascular endothelial growth factor (VEGF)

The VEGF is the major pro-angiogenic molecule. It has a key role in regulating physiological as well as pathological angiogenesis [120, 121]. Up to now, four genes structurally related to VEGF have been identified. Among their known products, there are placenta growth factors (PGF) [122–124], VEGF-a, VEGF-b [124], VEGF-c [125],VEGF-d [126] and VEGF-e [126]. The VEGF-a isoform is the most important VEGF molecule contributing to MM since its receptor, VEGFR-2, is highly expressed on plasma cells and endothelial cells in MM [127, 128]. After stimulation of VEGFR-2, mitogen-activated protein (MAP)-kinase is phosphorylated, resulting in an enhanced cell proliferation. Probably there is a paracrine mechanism behind the VEGF-induced stimulation of the endothelium: the plasma cell secretes VEGF-a, which binds to the VEGFR-2 on angiopoietic cells, ultimately activating a proliferative signal.

VEGF-a is also an important communication signal between stromal and myeloma cells, inducing the microenvironment to produce the same VEGF, but also other proangiogenic factors, such as FGF-β and TGF-β [129]. Furthermore, VEGF-a has an autocrine effect on the neoplastic cell, mediated by VEGFR-2 and the intracellular transmission pathway RAF-1/MEK-1, which would strengthen further angiogenic stimulus [129].

In tumors, hypoxia may be a consequence of the distance of the growing tumor cells from existent capillaries or inefficiency of the new vessels [130, 131]. Therefore, the expression of VEGF and its receptor is induced in angiopoietic cells in order to avoid cell death. Apart from the stimulation of neovascularization and the increase of production of cytokines and proteolytic enzymes contributing to neo-angiogenesis, VEGF leads to upregulation of some proto-oncogenes typically found in MM, such as V-ras, K-ras, V-raf, Src and Fos [132]. Indeed, inhibition of VEGFR-2 and VEGF-a reduces myeloma clone proliferation. Patients with MM have low levels of semaforina III, which has a physiological antiangiogenic effect, balancing the action of VEGF [133]. Microvascular density of the bone marrow assessed with anti CD34 and DC15 (which are antigens present on the endothelium) is increased in patients with MM compared to healthy controls and also seems to correlate with the stage of disease. Of note, although bone marrow vascularization appears to be a prognostic marker, it does not change after therapy [134]. Another important question is related to the measurable levels of the signaling proteins as well as their receptors. For example, Dales et al have demonstrated an increased expression of VEGF-R1 in diseases such as breast cancer, a disease in which VEGF-R1 seems to be a negative prognostic marker with a high risk of metastases and relapses [135] and the use of antibodies against VEGF-R1 determines a powerful inhibition of neoplastic cell growth [136].

Overall, drugs targeting mechanisms involving the various VEGF forms are promising targets for new treatment approaches. For example, Bevacizumab targets and blocks VEGF and VEGF's binding to its receptor on the vascular endothelium [137] and has demonstrated to be effective when used alone, and in combination with radiation in earlier preclinical studies [137, 138]. It is currently being studied clinically in many solid and blood tumors including primary systemic amyloidosis and MM [139, 140]. NCI's Cancer Therapy Evaluation Program sponsored a phase II study of bevacizumab plus thalidomide in MM. The study was closed early due to poor accrual. Combination therapy, in this limited sample, yielded similar results to single agent thalidomide. The small number of patients prevents correlation of VEGF or VEGFR1/VEGFR2 expression with response [141].

### Angiopoietins (Ang)

Angs, consisting of 4 structurally related proteins, termed Ang-1, 2, and 4, are ligands for the vascular-specific tyrosine kinase TIE-2 receptor located on endothelial cells. Ang-1 and Ang-4 behave as activating ligands for Tie-2, whereas Ang-2 and Ang-3 function as competitive antagonists. When bound to Tie-2, Ang-1 stimulates maturation and stabilization of the vascular wall [142, 143]. In contrast, Ang-2 antagonizes Tie-2 binding and induces vessel destabilization, leading to the angiogenic sprouting [144]. In MM Ang-1 and Ang-2, along with VEGF, possess an important role in the initiation of tumor angiogenesis [145]. This is further confirmed by the observation that tumor angiogenesis can be prevented using both antibodies against Ang-1 and VEGF-a, while neutralizing only either VEGF-a or Ang-1 reduces it partially [146]. Ang-2 serum levels, alone or in ratio with Ang-1, are importantly prognostic for response to therapy and mainly for survival [147]. These data highlight the role of the angiopoietins pathway in the biology of MM, which could be an interesting target for new anti-myeloma agents.

### Platelet-derived growth factor -beta (PDGF-β)

PDGF-beta is able to up-regulate the expression of the protein c-MYC, thus reducing the sensibility of cancer cells to melphalan treatment. Additionally, melphalan-resistant patients have an overexpression of c-MYC and higher levels of *PDGF-*β [148]. The latter correlated with disease burden (estimated by Durie-Salmon staging system, levels of IL-6 and beta-2-microglobulin) [149, 150]. Therefore, compounds targeting this mechanism could improve MM treatment. PTK787/ZK22258 (PTK/ZK) is a potent tyrosine kinase inhibitor of both vascular endothelial growth factor receptor 1 (VEGF-R1) and VEGF-R2, and also inhibits the tyrosine kinase activity of PDGFR-β, Flt-4, c-kit and c-fms, although with less potency [133]. PTK/ZK inhibits endothelial cell migration and proliferation without cytotoxic or antiproliferative effects on cells that do not express VEGF receptors [133]. Oral administration of PTK/ZK at a dose of 25-100 mg/kg/day decreases tumor growth in human cancer xenografts [133, 151]. Overall, PTK/ZK inhibits multiple essential signaling pathways involved in proliferation and fibrogenesis [152], which is why this molecule could improve the efficacy of existing MM treatments.

### Fibroblast growth factor beta

FGF-beta is able to trigger neovascularization in MM bone marrow [153]. In the altered micro-environment, the myeloma cells appear to be the main source of FGF-beta, which is why their expression levels could correlate with disease burden and therefore could be an important prognostic parameter for MM [154]. In fact, the concentration of FGF-beta is considerably increased in plasma cell lysates in the bone marrow and in peripheral blood of myeloma patients [154].

Apart from the pro-angiogenic effects of this growth factor, paracrine interactions between tumor cells and BMSC were observed: stimulation of stromal cells with FGF-beta induces an increase of IL-6 secretion. This process can be completely abrogated by the administration of antibodies against FGF-beta. On the other hand, stimulation with IL-6 increases the expression and secretion of FGF-beta by the myeloma cell lines [155].

### Matrix metallopeptidase (MMP)-1, MMP-2 and MMP-9

The expression of these metal proteinases is considerably increased in myeloma plasma cells as well as cells of the tumor environment, such as macrophages, fibroblasts and osteoblasts [156, 157]. Up to now their role in the context of tumor angiogenesis is unclear, but they probably are mediators for VEGF and PDGF-beta-dependent mechanisms. Indeed, *in vivo* studies in murine models indicate that zoledronic acid is able to inhibit the expression of MMP-9 of macrophage origin and finally neoangiogenesis [158].

Increased levels of the MMP-1 has been associated with an enhanced capacity to degrade collagen I [159]. In MMP-1 transgenic mice, the synthesis of the latent form of collagenase leads to extracellular matrix degradation [160]. MMP-2 instead directly modulates tumor invasion [161], favoring the spreading of myeloma cells. The mechanisms responsible for the upregulation of MMP activity remain to be elucidated.

Overall, the most important mechanisms in MM are the production of MMP-9, activation of MMP-2 and induction of MMP-1 by the neoplastic cells, since they are major players for bone resorption and tumor spreading. Some inhibitors of MMPs are now available. In arthritis, these inhibitors lead to a dramatic suppression of cartilage and bone destruction [162]. In cancer, the MMP inhibitor Batimastat was effective in a variety of murine tumor metastatic models and was successfully used in patients with malignant ascites [163] and malignant pleural effusion [164].

## OTHERS FACTORS

### Granulocyte colony stimulating factor (G-CSF)

G-CSF is a hematopoietic growth factor with several similarities to IL-6 [165, 166]. Both IL-6 and G-CSF induce the activation of NF-IL-6, which is involved in the synthesis of the IL-6 [167]. *In vivo* G-CSF appears to be a potent growth factor for myeloma cells [9]. Despite their well-known physiological functions, G-CSF and IL-6 could modulate neutrophils in bone marrow, altering the activation potential of signaling pathways in neutrophils, especially that of STAT3. Co-stimulation with G-CSF and IL-6 induced a higher level of phospho-STAT3 in neutrophils, stimulating angiogenesis and tumor growth [167].

### IL-10

IL-10 is another important growth factor contributing to the pathogenesis of MM. IL-10 has been proven to inhibit various immune functions, such as macrophage activation, cytokine production, and antigen presentation [168], while it induces both plasma cell proliferation and angiogenesis in MM [169]. IL-10 is also involved in initiating and promoting other malignancies [170].

High levels of IL-10 secreted by T regulatory cells or produced from myeloma cells can modulate antitumor host immune response, including the abrogation of DCs function [171, 172]. Furthermore, IL-10 seems to induce chemo-resistance [173, 174]. In particular, the serum concentration of IL-10 has been found much higher in MM patients than in normal healthy people, correlating with poor prognosis [175] and poor treatment response [175].

## CONCLUSIONS

The herein presented data provide evidence that the bone marrow environment is a very important player in various biological processes of myeloma development. The involved mechanisms are very complex and not only limited to osteoblast and osteoclast dysfunction, but include pathologic immune processes as well as growth factor dysregulations. Thus, these findings are the basis for innovative treatment approaches. Clinical trials designed to target immune and stromal components of the micro-environment are ongoing and, in combination with other anti-myeloma agents, should get us closer to our ultimate objective of eradicating the disease.
